# Analysis of circulating tumor cells from lung cancer patients with multiple biomarkers using high-performance size-based microfluidic chip

**DOI:** 10.18632/oncotarget.14203

**Published:** 2016-12-26

**Authors:** Wanlei Gao, Haojun Yuan, Fengxiang Jing, Shan Wu, Hongbo Zhou, Hongju Mao, Qinghui Jin, Jianlong Zhao, Hui Cong, Chunping Jia

**Affiliations:** ^1^ State Key Laboratory of Transducer Technology, Shanghai Institute of Microsystem and Information Technology, Chinese Academy of Sciences, Shanghai 200050, China; ^2^ Center of Laboratory Medicine, Affiliated Hospital of Nantong University, Nantong, Jiangsu 226000, China; ^3^ University of Chinese Academy of Sciences, Beijing 100039, China

**Keywords:** lung cancer, circulating tumor cells, mesenchymal marker, metastasis, size-based microfluidic chip

## Abstract

Circulating tumor cells (CTCs) have attracted pretty much attention from scientists because of their important relationship with the process of metastasis. Here, we developed a size-based microfluidic chip containing triangular pillar array and filter channel array for detecting single CTCs and CTC clusters independent of tumor-specific markers. The cell populations in chip were characterized by immune-fluorescent staining combining an epithelial marker and a mesenchymal marker. We largely decreased the whole time of detection process to nearly 1.5h with this microfluidic device. The CTCs were subsequently measured in 77 patients with lung cancer and 39 healthy persons. The microfluidic device allowed for the detection of CTCs with apparent high sensitivity and specificity (82.7% sensitivity and 100% specificity). Furthermore, the total CTC counts were found to be elevated in advanced patients with metastases when compared with those without (20.89±14.57 vs 8.428±5.858 cells/mL blood; P<0.01). Combined epithelial marker and mesenchymal marker analysis of CTCs could provide more information about metastasis in patients than only usage of epithelial marker. In conclusion, the development of the size-based microfluidic device for efficient capture of CTCs will enable detailed characterization of their biological properties and values in cancer diagnosis.

## INTRODUCTION

Lung cancer is among the most common and deadly cancers worldwide. Metastatic disease results in a large number of cancer related deaths. Most of lung cancer patients are diagnosed with advanced disease, and some with early stage disease have high recurrence rates [1].

During the metastatic process, primary tumor cells experience a series of steps to spread the disease from its original residing site to distant organs of the human body. Initially, tumor cells disseminated from solid tumors undergo epithelial-to-mesenchymal transition (EMT) for achieving their migratory and invasive properties [2]. Circulating tumor cells(CTCs) are tumor cells turning into blood circulation system, which are critical for metastasis [3]. It reported that CTC counts have a correlation with prognosis and progress of many metastatic diseases, such as breast, colon, prostate and lung cancers [4–7]. Therefore, quantification and characterization of CTCs can provide important clinical information for patients with metastatic cancer, thereby offering potential to design effective and individualized cancer therapies.

The large challenge of CTC detection is that CTCs are extremely rare in whole blood, even as low as 1 CTC per 10^7^ hematologic cells [8, 9]. Development of an effective enrichment process has become critical for detecting CTCs. In recent years, many technologies to effectively capture CTCs from blood have proven useful [1]. The magnetic bead labeled with antibodies against epithelial cell adhesion molecule (EpCAM) CTC detecting methods are widely used. Among them, CellSearch (Janssen Diagnostics, LLC, USA) system is the only platform approved by the US Food and Drug Administration(FDA) for clinical utility in metastatic colorectal, prostate and breast cancer [10]. In recent years, some methods based on immune-affinity or nanomaterials have been developed [11–13]. The capture efficiency of these methods heavily depends on EpCAM expression. However, the heterogeneity of EpCAM expression exists in different tumor cells, and it is even absent in some non-epithelial tumors [14]. To overcome the problem, new CTC isolation methods based on cells’ physical properties (i.e. density, size, mechanical plasticity and dielectric properties) have been developed. For example, ScreenCell® device is a size-based commercialized CTC isolation system, which is using polycarbonate filters [15]. However, the track-etched filters have the disadvantages of low porosity and random distributed pores, resulting in low efficiency and clogging. We designed a size-based filtration microfluidic chip previously [16], with the advantages of high capture efficiency, low production cost and ease handling. This size-based filtration microfluidic method in this study was improved for good application in blood sample.

After separation and enrichment, CTCs can be characterized with the broad spectrum of methods such as immune-cytological methods, fluorescent in situ hybridization or PCR adapted to single-cell analysis [17–20]. The most frequently used markers are EpCAM and different types of cytokeratins (CKs). While Fehm T et al. found that many CTCs are cytokeratin-negative [21]. The common antibodies lack the capability to detect some CTCs, which are mesenchymal in origin [22]. Therefore, simultaneous staining with epithelial marker and mesenchymal marker could provide more valuable information for CTC detection.

In this report, we presented a microfluidic chip incorporating triangular pillar array and filter channel array that can separate heterogeneous cells with marker for CTCs. Next, we analyzed a clinical test for detection of CTCs from lung cancer patients and healthy persons. We demonstrated that it can capture both CTCs with epithelial phenotype and those in the process of EMT. The data reported here hold great promise for the detection of CTCs from lung cancer patients, thus making Vimentin as a CTC marker.

## RESULTS

### Design and profile of the size-based microfluidic chip

**Figure 1:**
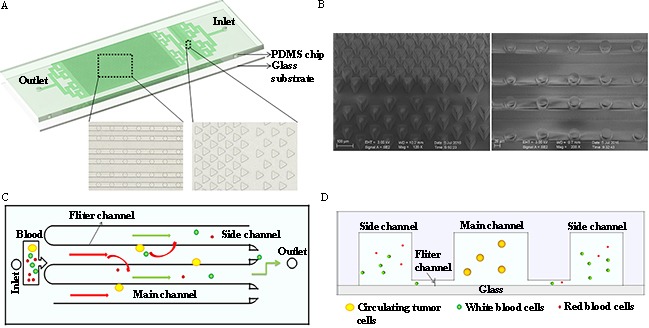
The design and operation of the size-based microfluidic device **A**. Schematic representation of the microfluidic device. The insets show the structure of the bulk filtration area and single cell filtration area under the microscope. **B**. SEM micrographs of triangular pillar arrays (left) in the bulk filtration area and channel array in the single cell filtration area (right). Isolation principle inside the device in vertical view **C**. and in profile view **D**.

A size-based microfluidic chip for CTC enrichment was designed with SolidWorks CAD design software (Waltham, MA, USA) as shown in Figure 1A. The device is composed of two filtration parts, bulk filtration area and single cell filtration area. The bulk filtration area is connected with the inlet via branch pipelines. The fundamental building block of the bulk filtration area is formed by three triangular pillars. Two triangular pillars constitute a filter funnel, where the edge of the third triangular pillar is placed to form a furcation. The first and second grade of narrowing channels’ width is 50μm and 20μm, respectively (Figure 1B. left). As sample flows, single blood cells or single CTCs pass through one of the two narrow channels at the furcation and entry to the single cell filtration area. In contrast, cell-clusters and bulk contaminants are held by the leading edge of the triangular pillar under a dynamic force balance. The bulk filtration area specifically captures cell clusters and does not trap single cells and this specificity enables clog-free processing of blood samples.

The single cell filtration area includes 30 main channels and 31 side channels. A row of filter channels is positioned between each main channel and contiguous side channel (Figure 1B. right). In addition, each main channel is connected to the outlet via a filter channel. The entire area is supported by rows of cylinders, which are located at filter channels. The interval distance and diameter of every cylinder in a row is 100μm and 40μm, respectively. Typical dimensions used in the device are as follows: main micro-channel, 80μm (width)×50μm (height), side micro-channel, 50μm (width)×50μm (height), and filtration micro-channel, 40μm (width)×10μm(height). The isolation strategy of the microfluidic chip is depicted in Figure 1C and 1D. As sample flows into the single cell filtration area, cells entry the main channels because the leading end of the side channels are blind. Most of the hematologic cells can pass through the filter channels into the side channels and then be eliminated from the outlet. However, the large size cells like CTCs are trapped at the main channel. In this study, the entire process of CTC isolation, staining and detection was conducted in one chip. The operation procedure is summarized in Table 1. 10min was long enough to filter 2mL blood sample.

**Table 1:** Operation program of size-based microfluidic device for the rapid detection of CTCs

no.	operation	state	volume	time
1	sample filtration	flow	2mL	10min
2	washing	flow	500μL	1min
3	permeabilization	incubation(RT)	20μL	10min
4	washing	flow	200μL	20sec
5	blocking	incubation(RT)	20μL	5min
6	staining	incubation(37°C)	20μL	40min
7	washing	flow	400μL	40sec
8	analyzing	Under microscopy		20min
Total detecting Time				87min

Abbreviation: RT (room temperature)

### Chip optimization and performance

**Figure 2:**
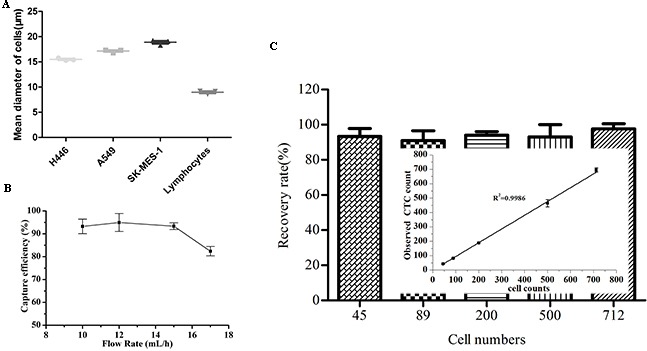
Spiked cancer cell lines test for capture rates **A**. Three repeated measurements for the mean diameter of H446, A549, SK-MES-1 and normal lymphocytes. **B**. Plot of H446 cell recovery with varying flow rate. **C**. Recovery rate vs. input cell number. H446 cells, ranging from 45,89,200,500,712 in total number, were spiked into 2mL of blood.

Firstly, we measured the sizes of three lung cancer cell lines and lymphocytes. The mean diameters of H446, A549, SK-MES-1 and normal lymphocytes were 15.49±0.25, 17.16±0.30, 18.88±0.50 and 8.97±0.35μm, respectively (Figure 2A). So 10μm height was applied in filter channel. To assess the performance of the device, we spiked the smallest cells (H446) into samples from healthy persons and captured them using the size-based microfluidic chip. The recovery rate maintained at above 90% for flow rates up to 15mL/h (Figure 2B), leading us to select 15mL/h for subsequent experiments. To evaluate the sensitivity of the device, H446 cells spiked into whole blood at different concentrations were recovered using the device. The overall recovery efficiency of the cancer cells was above 94%, which showed a potential clinical utility. (Figure 2C).

### The potential to harvest CK negative CTCs using the size-based microfluidic chip

**Figure 3:**
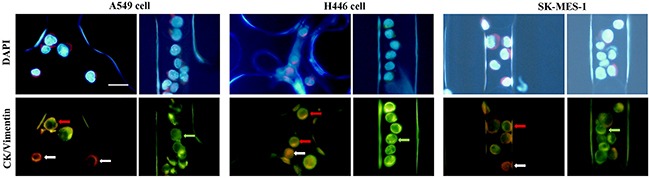
EMT expressions on A549, H446, SK-MES-1 cell lines Various types of signals combination were presented including cells purely expressing CK without Vimentin (green arrow), purely expressing Vimentin without CK (white arrow), expressing Vimentin and CK (red arrow). Scale bars 20mm.

To better understand the expressions of CK and Vimentin, we analyzed protein expression profiles of lung carcinoma cell lines (H446, SK-MES-1 cells and A549). The existence of EMT was tested in lung cancer cells by the three color immune-fluorescent staining (CK, CD45 and Vimentin) with the size-based microfluidic chip. Representative images are shown in Figure 3. The expression of CK and Vimentin were heterogeneous on those cells, with 17.16% of SK-MES-1 cells completely absent of CK expression (16.12% for H446 cells, 14.62% for A549 cells). Moreover, 10.06 % of SK-MES-1 cells,13.12% H446 cells and 10.53% A549 cells expressed Vimentin but not CK, indicating the existence of EMT (Table 2). Common marker CK would lack the ability of identifying some cells expressing more mesenchymal proteins.

**Table 2:** The proportion of lung cancer cells expressing epithelial and mesenchymal makers

Cell lines	CK+/Vimentin- number(%)	CK+/Vimentin+ number(%)	CK-/Vimentin+ number(%)	CK-/Vimentin-number(%)	Total number
SK-MES-1	112(66.27)	28(16.57)	17(10.06)	12(7.10)	169
H446	95(59.38)	38(23.75)	21(13.12)	6(3.75)	160
A549	104(60.82)	42(24.56)	18(10.53)	7(4.09)	171

### Comparison of data obtained from the size-based microfluidic chip and EpCAM-based method

**Figure 4:**
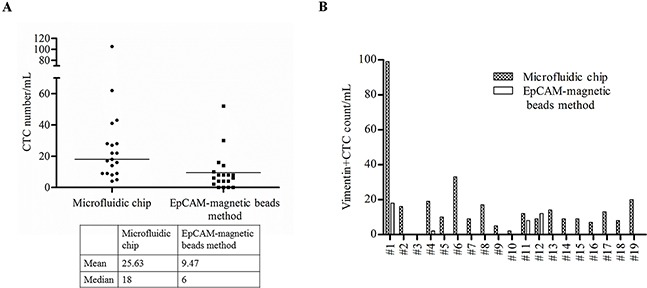
CTC detection by the size-based microfluidic chip and EpCAM-magnetic beads method **A**. Comparison of CTC counts in lung cancer patients using the size-based microfluidic chip and EpCAM-magnetic beads method. **B**. Comparison of Vimentin+/CD45- CTC counts of the same lung cancer patients’ sample using the two methods.

We compared the performance of the size-based microchip against the EpCAM-based method for CTCs detection in 19 blood samples. Using the EpCAM-based method, 15 patients had detectable CTCs defined ≥1 CTC (Figure 4A). CTC/mL detected by the size-based microchip vs the EpCAM-based method revealed that the mean CTC/mL was 25.63 vs 9.47, the median CTC/ mL was 18 vs 6 and that the range was 4-105 vs 0-52, respectively. The results indicated that the CTC counts were significantly different between the two methods. Vimentin+/CD45-CTC counts obtained by the two approaches is shown in Figure 4B. Only in 4 patients could we nicely isolate mesenchymal CTCs using the EpCAM-based method. The majority of patients (18/19) detected by the size-based microfluidic chip had ≥1 Vimentin+CD45-cells/mL, highlighting the efficiency of CTC isolation by our device.

### Application of the size-based microfluidic device in lung cancer patients

**Figure 5:**
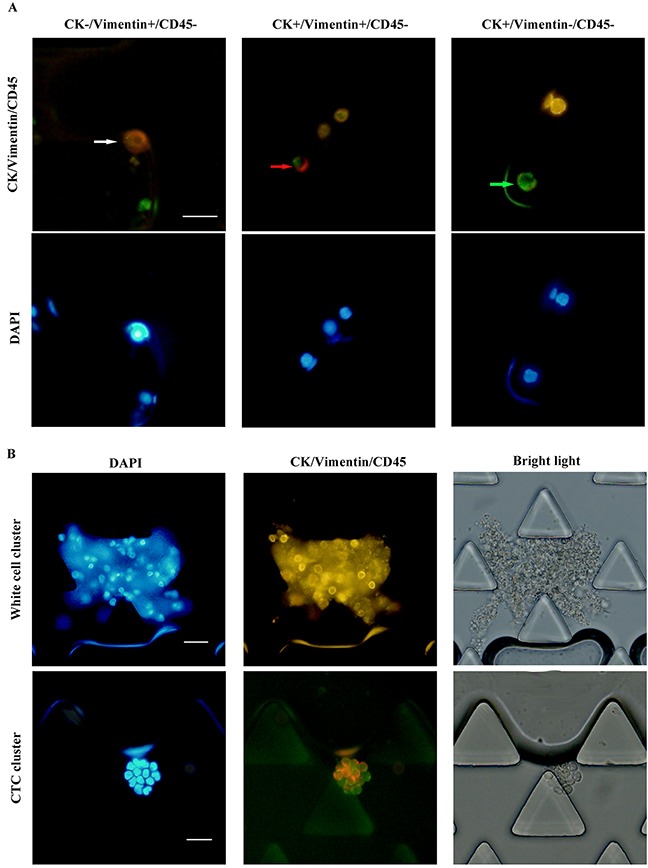
**A**. Representative images for different types of detected cells in lung cancer patients including CK-/Vimentin+/CD45- cell (white arrow); CK+/Vimentin+/CD45- cell (red arrow); CK+/Vimentin-/CD45- cell (green arrow). **B**. Representative images of CK-/Vimentin-/CD45+ cell cluster (up) and CTC cluster(down) staining with CK(green), Vimentin (red), CD45 (orange) and DAPI (nuclei, blue) isolated by the triangular pillar array. Scale bars 20μm.

**Figure 6:**
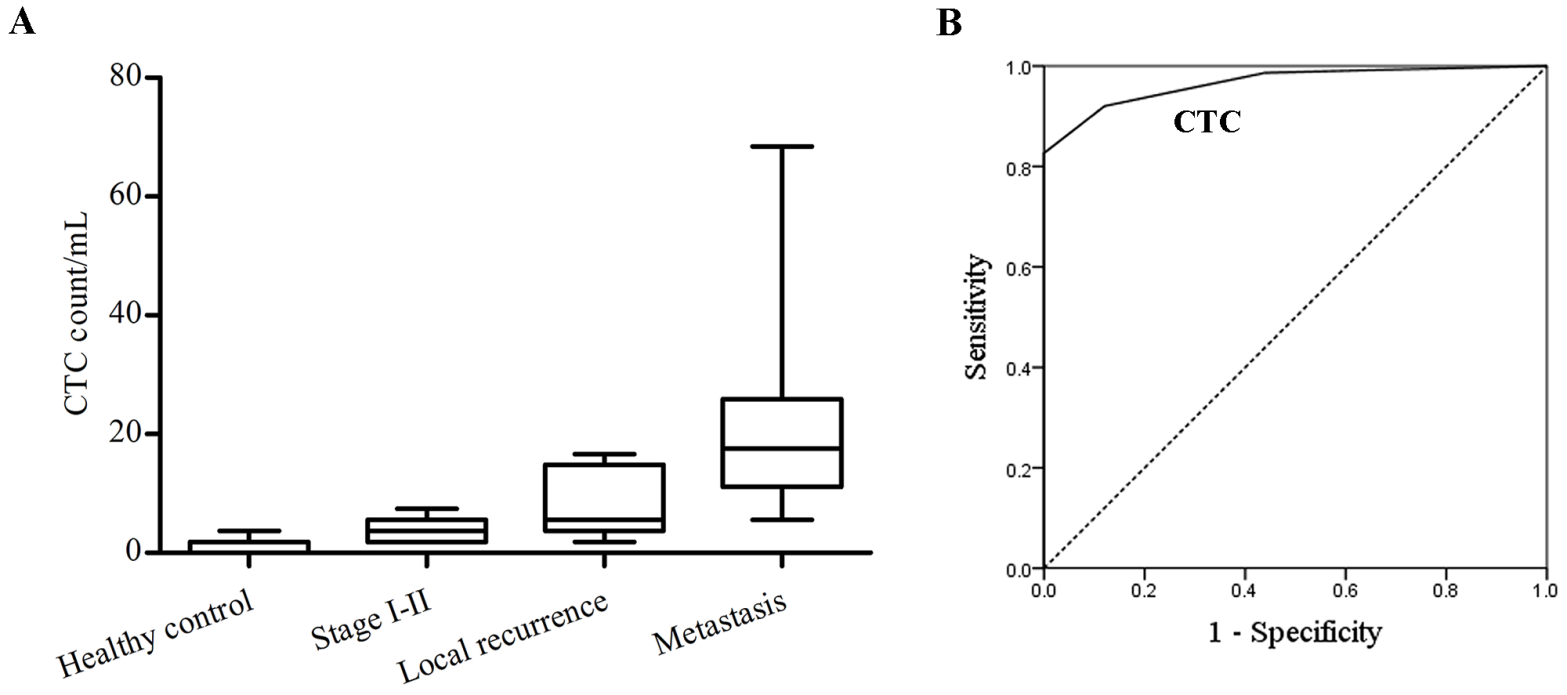
Distribution of CTC levels and ROC curve analysis **A**. Distribution of CTC counts in healthy donors and in patients with lung cancer. The box plot demonstrates the median, lower and upper quartiles (25^th^, 75^th^ percentiles). **B**. ROC curve for CTC counts to discriminate patients from healthy persons.

The performance of the size-based microfluidic device in detecting cancer patients’ CTCs was assessed by testing the blood samples from 77 lung cancer patients and 39 healthy donors. Clinical data of the patients are shown in Table 3. Analyses of lung cancer blood samples revealed that three cell types of CK+/Vimentin-/CD45-, CK+/Vimentin+/CD45- and CK-/Vimentin+/CD45-CTC were existed in the blood of cancer patients (Figure 5A). Some CD45+cell clusters and CTC clusters were also captured at the bulk filtration area in the chip (Figure 5B). As demonstrated in Figure 6A, CTCs were successfully enriched in 77 specimens with a mean number of 15.16 /mL (range of 1.85-68.45), whereas CTCs were observed in 17 out of the 39 healthy volunteers, with a mean CTC count of only 0.95 /mL (range of 0-3.7). Lung cancer patients had significantly higher CTC count than healthy persons.(P<0.0001). In addition, CTC counts of patients with distant metastases were marked higher than stage I-II patients or those with local recurrence (21.50±14.78vs. 4.10±2.30vs. 11.72±5.15, P<0.05). The significant difference in CTC level was also found in local recurrent patients and stage I-II patients, with more CTCs detected in local recurrent patients than stage I-II patients (P<0.05). These findings revealed that CTC numbers were relevant to tumor stage and disease progression.

**Table 3:** Basic characteristics for the cancer patients subject to CTC analyses

Patient characteristics	Lung cancer
Number of patients	77
Age	
Mean	62.7
Median(range)	64(26-88)
Gender	
Male	57
Female	20
Pathological type	
squamous cell carcinoma	18
adenocarcinoma	38
small cell lung cancer	6
NOS	15
Stage	
Newly diagnosed	
stageI-II	23
Local recurrence	9
Metastasis	45

Abbreviation: NOS (not otherwise specified)

The optimum cut-off level for CTC to discriminate between healthy persons and lung cancer patients was 4.6 /mL (AUC-ROC was 0.966, 95%CI, 0.936-0.997, with a sensitivity of 82.7% and specificity of 100%, P=0.000; see Figure 6B). The ROC curve analysis therefore demonstrated 4.6 /mL as a potential value for the diagnosis of lung cancer using this device.

### CTC counts relate to metastatic status

**Figure 7:**
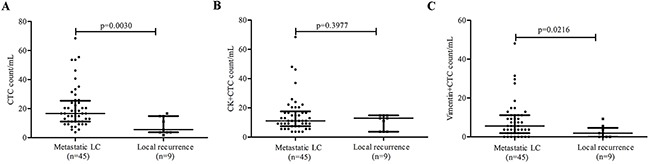
Correlation between CTC subtype counts and metastatic lung cancer (LC) Plots are shown for **A**. total CTC counts, **B**. CK+CTC (CK+/Vimentin-/CD45-CTC plus CK+/Vimentin+/CD45-CTC) counts, **C**. Vimentin+CTC (CK+/Vimentin+/CD45-CTC plus CK-/Vimentin+/CD45-CTC) counts in 1mL blood. Lines represent the interquartile range and median value.

We further analyzed 54 lung cancer patients with advanced disease to test the relation between CTC subsets and metastatic status. The pool included 9 patients with local recurrence not metastasis and 45 patients with metastatic lung cancer. When examining the total CTC count (CK+/Vimentin-/CD45-CTC plus CK+/Vimentin+/CD45-CTC plus CK-/Vimentin+/CD45-CTC), a significant difference was found between metastatic and non-metastatic disease (20.89±14.57 vs 8.428±5.858 / mL blood; P=0.0030) (Figure 7A). However, the CK+CTCs (CK+/Vimentin-/CD45-CTC plus CK+/Vimentin+/CD45-CTC) subset alone cannot distinguish metastatic from non-metastatic disease (15.24±12.72 vs 10.28±5.076cells/mL; P=0.3977) (Figure 7B). In contrast, Vimentin+CTC (CK+/Vimentin+/CD45-CTC plus CK-/Vimentin+/CD45-CTC) counts remained significantly different (8.463±9.686 vs 2.467±3.204 cells/mL; P=0.0216) (Figure 7C). Overall, combined epithelial marker and mesenchymal marker analysis of CTCs could provide more information about metastasis in patients with lung cancer than only usage of epithelial marker.

## DISCUSSION

In recent years, CTCs have gained much attention in the field of cancer research serving as a functional biomarker and as a potential tool in the study of metastasis. Analysis of CTC is a minimally-invasive approach that can help with clinical diagnosis, guide therapeutic selection and monitor prognosis. So the most important point is establishing a reliable CTCs analyzing strategy.

Many CTC detecting methods have been developed with variable extent of success. In this study, we further optimized the size-based microfluidic chip that had previously reported by our group [16]. The principle behind this microfluidic device is that most cancer cells are larger size and less deformability than blood cells make them less likely to traverse through the channels while most blood cells pass through [23]. The size-based microfluidic device presented here thus encompasses major advances over the previous methods. While very easy to use and time-saving, our size-based microfluidic device allows CTC isolation with high sensitivity and specificity (82.7% sensitivity and 100% specificity), and has the capability of identifying tumor cells in the same device. The main channels in the previous device sometimes were clogged because of some cell bulks in blood, which decreased the throughput and speed. We designed a bulk filtration area in front of the body area to prevent the cell clusters into the single cell filtration area. The triangular pillar arrays in the bulk filtration area captured cell clusters independent of their deformability [24]. Captured clusters were trapped under a dynamic force balance and single cells passed into next area. We improved the flow rate from 0.5 mL/h to 15 mL/h and the capture efficiency still remained 94%. Only 10 min were required to process 2 mL sample, which significantly reduced the detection time compared to previously reported methods. So the total time of less than 1.5h, was quicker than CellSearch systems (4h in total for CTC isolation). Therefore, these modifications of the structure and process make the size-based microfluidic device efficient for clinical use.

CTCs identification are mainly based on biological properties, with antibodies against tumor-associated antigen (positive marker) and the common leukocytes antigen (negative marker). Although proved to be diagnostically useful, EpCAM and different types of CKs are, however, discussed to be insufficient to detect the entire population of CTCs in blood [14, 25]. The yields of CTCs identified with these sets of markers are frequently poor even in some highly aggressive types of tumors (e.g. normal like breast cancer, ovarian cancer and pancreatic adenocarcinoma). [26, 27]. Some reports found that the presence of mesenchymal markers on CTCs has strongly associated with worse prognosis than those with the expression of CK alone, indicating that common identification based on epithelial marker may omit the most aggressive CTC-subset [28, 29]. Vimentin has been regarded as a novel marker, with preferentially expressed in moderately and well-differentiated lung adenocarcinomas [30]. When analyzed by immune-fluorescent staining, EMT-related markers are frequently co-expressed with different CKs [31, 32]. Therefore, in addition to CK, Vimentin was selected for identifying CTCs in this study.

Using three-color immune-fluorescent staining, we obtained high definition images of immune-stained cells in all 3 kinds of lung carcinoma cell lines(H446, SK-MES-1 cells and A549). In H446 cells, 13.12% cells were absent for CK but Vimentin-positive. In A549 and SK-MES-1 cells, we also found 10.53% and 10.06% CK-/Vimentin+/CD45- cells. These results demonstrated the common used standard to identify CTCs may result in false negative. While cell lines do not effectively reflect CTCs in a natural biological fluid, especially in terms of heterogeneity [33]. CK+/Vimentin+/CD45- and CK-/Vimentin+/CD45- cell populations were also detected in blood samples from lung cancer patients with our method in addition to CK+/Vimentin-/CD45- cells. Those cells co-expressing CK and Vimentin may be CTCs undergoing EMT. Further investigation based on clinical samples should be conducted to determine whether Vimentin+/CD45- cells is related to clinical features and outcomes.

We have compared the detection of CTCs between the size-based microfluidic chip and the EpCAM-magnetic bead based method in 19 lung cancer patients. Among 18 samples, the microfluidic chip detected more Vimentin+/CD45- CTCs than that with the EpCAM-magnetic bead based method. These results demonstrated that a subpopulation of cells which expressed low EpCAM was missed by the anti-EpCAM antibody based method. Thus, the size-based microfluidic device displayed increased sensitivity for patients with low numbers of CTCs, which may express Vimentin

In this work, we continued our study to investigate the prognostic value of lung cancer CTCs. The results indicated that CTC counts were marked higher in lung cancer patients than in healthy persons and related to tumor stage. There were increased CTC counts in patients with metastases compared with stage I-II patients or those with local recurrence. In addition, the cut-off value of this method is higher than that obtained by commonly used methods [34, 35]. We explain that other commonly used methods based on epithelial antibodies may fail to detect CTCs undergoing EMT. In the sample from lung cancer patients, this method can capture clusters of over three tumor cells, which may provide more promising information for predicting patient prognosis. Because of the ability to detect these mesenchymal CTCs, we consider it a novel method to monitor metastatic progression that may have prognostic value. Correlation of CTCs and metastatic was further investigated. These results strongly suggested that Vimentin+CTC count in patients with metastatic lung cancer were significantly higher compared to those with no metastasis. This raises the possibility that combination of CK and Vimentin expressions may have perhaps prediction regarding progression of metastases. This initial observation also requires larger-scale trials.

In summary, we developed a novel and low cost size-based microfluidic device to enrich CTCs and combined mesenchymal maker and epithelial maker to identify subsets of CTCs. The new design of triangular pillar array and filter channel array in the chip achieved high-efficiency single cell and cell clusters enrichment. The microfluidic chip provided a high specificity and sensitivity for the qualification of CTCs while identifying protein expression phenotype. Although this method has been tested in a small clinical sample cohort, it has a great applied potential for rapid analysis of CTCs in clinical diagnosis and cancer prognosis prediction.

## MATERIALS AND METHODS

### Patients

The study was approved by the Affiliated Hospital of Nantong University, initiated on July, 2014. Informed and written consent was obtained from all patients and healthy donors. Clinical data were collected for age, gender and pathological diagnosis, prior treatment received and clinical outcomes. This study protocol was approved by the Ethics Review Committee of the Affiliated Hospital of Nantong University.

### Blood processing

Peripheral blood samples from patients were collected in Vacutainer tubes containing the anticoagulant EDTA. All peripheral blood specimens were stored at 4°C and processed within 96h. Blood samples were centrifuged 10 min at 2500 rpm, and then the precipitates were incubated with red blood cell lysis buffer (0.139M NH_4_Cl, 0.02MTris, pH7.2) for 45 min on ice. The supernatant was removed by 10 min centrifugation at 2500 rpm, and a further repeat of the incubation and centrifugation were performed until little red blood cells left. The cell pellet was resuspended in 1% paraformaldehyde in PBS buffer and subsequently incubated at room temperature for 30min. Following centrifugation 10 min at 2500, the supernatant was removed and the cell pellet was resuspended in PBS buffer.

### Size-based microfluidic chip fabrication

The device comprised of two layers: a PDMS microfluidic chip containing microchannel structures and a glass slide. A Si master with microchannel structures was fabricated using photolithography, silica wet etching and deep reactive ion etching (DRIE). Before PDMS molding, the Si master was exposed to trichloro-(1H,1H,2H,2H-perfluorooctyl) silane (Sigma-Aldrich, USA) vapor under vacuum overnight, in favor of the PDMS layer's release. Degassed PDMS (Sylgard 184, Dow Corning, Midland, Michigan, USA) at a base/curing agent ratio of 10:1(w/w) was poured over the Si mold and then cured for about 1 h at 65°C in an oven. After curing, the PDMS layer was peeled off the Si mold. At last, the PDMS layer was bonded by plasma treatment onto a clean glass slide, thereby resulting in a 2-layer microfluidic chip.

### Cell culture and spiking

The A549(derived from lung adenocarcinoma), SK-MES-1(derived from lung squamous carcinoma) and H446(derived from small cell lung cancer) cell lines were obtained from cell bank of Chinese Academy of Sciences. Prior to test, cells were spiked at a known concentration in PBS or healthy donor’ samples. Cells’ concentration and diameters were measured by Count star automated cell counter (Inno-Alliance Biotech, USA) following the manufacturer's instructions.

### Experimental setup and device operation

The strategy of enrichment of CTCs was essentially similar to one that we previously published, with some modifications [16]. Briefly, 2mL diluted samples were leaded into the chip by a syringe pump (PHD 22/2000, HAVARD apparatus, Massachusetts, USA). The syringe pump provided a constant negative pressure for the chip during operation. The blood samples were pumped from the inlet into the microfluidic chip for CTC enrichment and the small size cells such as erythrocytes and leukocytes were passed through the chip into the pump.

### Identification of CTCs

After the process of the sample, tumor cells were identified based on immunofluorescence assay. The immunofluorescence reaction was done directly in the chip. Firstly, the chip was rinsed with washing buffer (PBS containing 0.05%Tween). Subsequently, a solution of 0.2% Triton X-100 (PBS containing 0.2% Triton X-100) was loaded onto the device and incubated for 10 min to permeabilize the cell membranes. After that, the chip was cleaned with washing buffer and then treated with PBS containing 1% BSA for 5min. Then the captured cells were immuno-stained with anti-CD45 antibody conjugated to phycoerythrin (MEM-28, Abcam, UK), anti-CK antibody conjugated to isothiocyanate (C-11, Abcam, UK), anti-Vimentin antibody conjugated to Alexa Fluor^®^ 594 (EPR3776, Abcam, UK) and 4`-6-diamidino-2-phenylindole (DAPI; Beyotime Institute of Biotechnology, USA) for 40min at 37°C. Finally, the chip was rinsed with the washing buffer to remove the unbound antibody. The microchip was scanned with an inverted microscope (Model IX51; Olympus) linked with image analysis software (DP Controller; Olympus). The cells that stained CK+/Vimentin+/CD45-/DAPI+, CK+/Vimentin-/CD45-/DAPI+ and CK-/Vimentin+/CD45-/DAPI+ were scored as CTCs.

### CTCs detection by anti-EpCAM magnetic beads method

CTCs enrichment using the CELLection^TM^ Epithelial Enrich kit (Invitrogen, USA) was carried out according to the manufacturer's protocol. After enrichment, the bead-bound cells were permeabilized with a solution of 0.2%Triton X-100 for 10 min and fluorescently labelled with anti-CD45 antibody conjugated to phycoerythrin, anti-CK conjugated to isothiocyanate, anti-Vimentin conjugated to Alexa Fluor® 594 and DAPI for 40min at 37°C. After each processing step, captured cells were washed by wash buffer to remove excess reagents in a magnetic. Finally, the bead-bound cells were transferred directly onto a slide and identified in the method as above described.

### Statistical analysis

Statistical analyses were performed using SPSS software (version 16.0, SPSS Inc., Chicago, IL). Regression analysis was performed to assess the accuracy of tumor cells detection. A Mann-Whitney test were used in cases of two independent samples. All tests were two-sided and were performed at a 5% level of significance.

## References

[1] O'Flaherty JD, Gray S, Richard D, Fennell D, O'Leary JJ, Blackhall FH, O'Byrne KJ (2012). Circulating tumour cells, their role in metastasis and their clinical utility in lung cancer. Lung Cancer.

[2] Lee A, Park J, Lim M, Sunkara V, Kim SY, Kim GH, Kim MH, Cho YK (2014). All-in-one centrifugal microfluidic device for size-selective circulating tumor cell isolation with high purity. Anal Chem.

[3] Sonnenberg A, Marciniak JY, Rassenti L, Ghia EM, Skowronski EA, Manouchehri S, McCanna J, Widhopf GF, Kipps TJ, Heller MJ (2014). Rapid electrokinetic isolation of cancer-related circulating cell-free DNA directly from blood. Clin Chem.

[4] Aggarwal C, Meropol NJ, Punt CJ, Iannotti N, Saidman BH, Sabbath KD, Gabrail NY, Picus J, Morse MA, Mitchell E, Miller MC, Cohen SJ (2013). Relationship among circulating tumor cells, CEA and overall survival in patients with metastatic colorectal cancer. Ann Oncol.

[5] de Bono JS, Scher HI, Montgomery RB, Parker C, Miller MC, Tissing H, Doyle GV, Terstappen LWWM, Pienta KJ, Raghavan D (2008). Circulating Tumor Cells Predict Survival Benefit from Treatment in Metastatic Castration-Resistant Prostate Cancer. Clin Cancer Res.

[6] Tanaka F, Yoneda K, Kondo N, Hashimoto M, Takuwa T, Matsumoto S, Okumura Y, Rahman S, Tsubota N, Tsujimura T, Kuribayashi K, Fukuoka K, Nakano T, Hasegawa S (2009). Circulating Tumor Cell as a Diagnostic Marker in Primary Lung Cancer. Clin Cancer Res.

[7] Cristofanilli M, Budd GT, Ellis MJ, Stopeck A, Matera J, Miller MC, Doyle GV, Allard WJ, Terstappen LW, Hayes DF (2005). Presence of circulating tumor cells (CTC) in metastatic breast cancer (MBC) predicts rapid progression and poor prognosis. J Clin Oncol.

[8] Paterlini-Brechot P, Benali NL (2007). Circulating tumor cells (CTC) detection: Clinical impact and future directions. Cancer Lett.

[9] Tibbe AGJ, Miller MC, Terstappen LWMM (2007). Statistical considerations for enumeration of circulating tumor cells. Cytom Part A.

[10] Miller MC, Doyle GV, Terstappen LW (2010). Significance of Circulating Tumor Cells Detected by the CellSearch System in Patients with Metastatic Breast Colorectal and Prostate Cancer. J Oncol.

[11] Sun N, Wang J, Ji LY, Hong SN, Dong JJ, Guo YH, Zhang KC, Pei RJ (2015). A Cellular Compatible Chitosan Nanoparticle Surface for Isolation and In Situ Culture of Rare Number CTCs. Small.

[12] Sun N, Liu M, Wang J, Wang Z, Li X, Jiang B, Pei R (2016). Chitosan Nanofibers for Specific Capture and Nondestructive Release of CTCs Assisted by pCBMA Brushes. Small.

[13] Sun N, Li XP, Wang ZL, Zhang RH, Wang JE, Wang KW, Pei RJ (2016). A Multiscale TiO2 Nanorod Array for Ultrasensitive Capture of Circulating Tumor Cells. Acs Appl Mater Inter.

[14] Mikolajczyk SD, Millar LS, Tsinberg P, Coutts SM, Zomorrodi M, Pham T, Bischoff FZ, Pircher TJ (2011). Detection of EpCAM-Negative and Cytokeratin-Negative Circulating Tumor Cells in Peripheral Blood. J Oncol.

[15] Desitter I, Guerrouahen BS, Benali-Furet N, Wechsler J, Janne PA, Kuang YA, Yanagita M, Wang LL, Berkowitz JA, Distel RJ, Cayre YE (2011). A New Device for Rapid Isolation by Size and Characterization of Rare Circulating Tumor Cells. Anticancer Res.

[16] Huang T, Jia CP, Jun Y, Sun WJ, Wang WT, Zhang HL, Cong H, Jing FX, Mao HJ, Jin QH, Zhang Z, Chen YJ, Li G, Mao GX, Zhao JL (2014). Highly sensitive enumeration of circulating tumor cells in lung cancer patients using a size-based filtration microfluidic chip. Biosens Bioelectron.

[17] Pantel K, Brakenhoff RH, Brandt B (2008). Detection, clinical relevance and specific biological properties of disseminating tumour cells. Nat Rev Cancer.

[18] Swennenhuis JF, Tibbe AG, Levink R, Sipkema RC, Terstappen LW (2009). Characterization of circulating tumor cells by fluorescence in situ hybridization. Cytometry A.

[19] Stoecklein NH, Klein CA (2010). Genetic disparity between primary tumours, disseminated tumour cells, and manifest metastasis. Int J Cancer.

[20] Hannemann J, Meyer-Staeckling S, Kemming D, Alpers I, Joosse SA, Pospisil H, Kurtz S, Gorndt J, Puschel K, Riethdorf S, Pantel K, Brandt B (2011). Quantitative High-Resolution Genomic Analysis of Single Cancer Cells. Plos One.

[21] Fehm T, Sagalowsky A, Clifford E, Beitsch P, Saboorian H, Euhus D, Meng SD, Morrison L, Tucker T, Lane N, Ghadimi BM, Heselmeyer-Haddad K, Ried T, Rao C, Uhr J (2002). Cytogenetic evidence that circulating epithelial cells in patients with carcinoma are malignant. Clin Cancer Res.

[22] Hyun KA, Goo KB, Han H, Sohn J, Choi W, Kim SI, Jung HI, Kim YS (2016). Epithelial-to-mesenchymal transition leads to loss of EpCAM and different physical properties in circulating tumor cells from metastatic breast cancer. Oncotarget.

[23] Vona G, Estepa L, Beroud C, Damotte D, Capron F, Nalpas B, Mineur A, Franco D, Lacour B, Pol S, Brechot C, Paterlini-Brechot P (2004). Impact of cytomorphological detection of circulating tumor cells in patients with liver cancer. Hepatology.

[24] Sarioglu AF, Aceto N, Kojic N, Donaldson MC, Zeinali M, Hamza B, Engstrom A, Zhu H, Sundaresan TK, Miyamoto DT, Luo X, Bardia A, Wittner BS (2015). A microfluidic device for label-free, physical capture of circulating tumor cell clusters. Nat Methods.

[25] Fehm T, Muller V, Alix-Panabieres C, Pantel K (2008). Micrometastatic spread in breast cancer: detection, molecular characterization and clinical relevance. Breast Cancer Res.

[26] Marth C, Kisic J, Kaern J, Trope C, Fodstad O (2002). Circulating tumor cells in the peripheral blood and bone marrow of patients with ovarian carcinoma do not predict prognosis. Cancer.

[27] de Albuquerque A, Kubisch I, Breier G, Stamminger G, Fersis N, Eichler A, Kaul S, Stolzel U (2012). Multimarker Gene Analysis of Circulating Tumor Cells in Pancreatic Cancer Patients: A Feasibility Study. Oncology-Basel.

[28] Gradilone A, Naso G, Raimondi C, Cortesi E, Gandini O, Vincenzi B, Saltarelli R, Chiapparino E, Spremberg F, Cristofanilli M, Frati L, Agliano AM, Gazzaniga P (2011). Circulating tumor cells (CTCs) in metastatic breast cancer (MBC): prognosis, drug resistance and phenotypic characterization. Ann Oncol.

[29] Konigsberg R, Obermayr E, Bises G, Pfeiler G, Gneist M, Wrba F, de Santis M, Zeillinger R, Hudec M, Dittrich C (2011). Detection of EpCAM positive and negative circulating tumor cells in metastatic breast cancer patients. Acta Oncol.

[30] Hofman V, Ilie MI, Long E, Selva E, Bonnetaud C, Molina T, Venissac N, Mouroux J, Vielh P, Hofman P (2011). Detection of circulating tumor cells as a prognostic factor in patients undergoing radical surgery for non-small-cell lung carcinoma: comparison of the efficacy of the CellSearch Assay (TM) and the isolation by size of epithelial tumor cell method. Int J Cancer.

[31] Armstrong AJ, Marengo MS, Oltean S, Kemeny G, Bitting RL, Turnbull JD, Herold CI, Marcom PK, George DJ, Garcia-Blanco MA (2011). Circulating Tumor Cells from Patients with Advanced Prostate and Breast Cancer Display Both Epithelial and Mesenchymal Markers. Mol Cancer Res.

[32] Lecharpentier A, Vielh P, Perez-Moreno P, Planchard D, Soria JC, Farace F (2011). Detection of circulating tumour cells with a hybrid (epithelial/mesenchymal) phenotype in patients with metastatic non-small cell lung cancer. Brit J Cancer.

[33] Gabriel MT, Calleja LR, Chalopin A, Ory B, Heymann D (2016). Circulating Tumor Cells: A Review of Non-EpCAM-Based Approaches for Cell Enrichment and Isolation. Clin Chem.

[34] Chen Q, Ge F, Cui W, Wang F, Yang Z, Guo Y, Li LY, Bremner RM, Lin PP (2013). Lung cancer circulating tumor cells isolated by the EpCAM-independent enrichment strategy correlate with Cytokeratin 19-derived CYFRA21-1 and pathological staging. Clin Chim Acta.

[35] Krebs MG, Sloane R, Priest L, Lancashire L, Hou JM, Greystoke A, Ward TH, Ferraldeschi R, Hughes A, Clack G, Ranson M, Dive C, Blackhall FH (2011). Evaluation and prognostic significance of circulating tumor cells in patients with non-small-cell lung cancer. J Clin Oncol.

